# Knockdown of circ-ABCB10 promotes sensitivity of lung cancer cells to cisplatin via miR-556-3p/AK4 axis

**DOI:** 10.1186/s12890-019-1035-z

**Published:** 2020-01-13

**Authors:** Zhihui Wu, Qiang Gong, Yan Yu, Jialin Zhu, Wencan Li

**Affiliations:** 1 Department of Thoracic Surgery, Zhuzhou Central Hospital, 116 Jiangnan Road, Tianyuan District, Zhuzhou City, 412007 Hunan Province China; 2 Genome Center, KingMed Diagnostics of Changsha, Zhuzhou City, 412007 Hunan Province China

**Keywords:** Circ-ABCB10, Cisplatin, miR-556-3p, AK4, Lung cancer

## Abstract

**Background:**

Due to the acquired drug resistance, the potency of cisplatin-based chemotherapy is limited in lung cancer, which is a big obstacle in clinical treatment of lung cancer. Abundant evidence has revealed that circular RNAs (circRNAs) exerted facilitating or suppressive function on the tumorigenesis of multiple cancers. The oncogenic role of circ-ABCB10 in breast cancer and clear cell renal cell carcinoma has been validated in recent researches. However, the regulatory mechanism of circ-ABCB10 and its relation to cellular sensitivity to cisplatin in lung cancer is poorly understood.

**Methods:**

The expression and characteristic of circ-ABCB10 were analyzed by RT-qPCR and nucleic acid electrophoresis. CCK-8, colony formation, TUNEL and transwell assays were applied to probe the role of FOXD3-AS1 in lung cancer. The interactions of miR-556-3p with circ-ABCB10 and AK4 were testified by luciferase reporter and RIP assays.

**Results:**

Circ-ABCB10 was markedly upregulated and featured with loop structure in lung cancer. Circ-ABCB10 depletion suppresses lung cancer progression and sensitizes lung cancer cells to cisplatin. Molecular mechanism assays manifested that circ-ABCB10 bound with miR-556-3p and negatively modulated miR-556-3p expression. Additionally, AK4 was testified to be the downstream target of miR-556-3p. More importantly, rescue assays clarified that upregulation of AK4 could reverse the cisplatin-sensitizing and tumor-suppressing effect of circ-ABCB10 knockdown on lung cancer cells.

**Conclusions:**

Circ-ABCB10 knockdown enhances sensitivity of lung cancer cells to cisplatin by targeting miR-556-3p/AK4 axis.

## Background

Lung cancer is diagnosed as the most prevalent malignancy globally, with high incidence and death rate. For lung cancer, nearly 1.8 million new cases are diagnosis and 1.6 million cases died each year, and the deaths triggered by lung cancer take up 19% of all cancer-associated death cases [1]. Known as a dominating cause of cancer-related death, lung cancer with a steady rise in occurrence rate has become a big obstacle for human health [2]. High rates of recurrence and metastasis have been referred to as the major factors contributing to poor prognosis of patients with lung cancer [3]. In spite of the improvement of diagnostic and therapeutic methods, the 5-year overall survival rate of patients suffering from lung cancer is less than 20% [4]. More importantly, resistance to lung cancer treatment is closely related to abnormal expression of oncogenic or anti-tumor genes, including changes in the biological features of malignancies, cell proliferation, metastasis, apoptosis, etc. [5]. Though cisplatin has already come into use as a kind of anti-cancer chemotherapy agent, multiple cancers (lung cancer included), may develop the acquired resistance to cisplatin, which is a stumbling block on the way to improving the efficacy of chemotherapy [6]. In addition, cisplatin cytotoxicity remains a prevalent side-effect of cisplatin [7]. Therefore, studying the mechanism underlying the cellular sensitivity to cisplatin in lung cancer is of extreme importance to enhance the efficacy of chemotherapy for lung cancer based on combined agents with specific molecular mechanisms.

Circular RNAs (circRNAs), microRNAs (miRNAs) as well as long noncoding RNAs (lncRNAs) belong to noncoding RNA (ncRNAs), among which circRNAs are featured with loop structure. Much attention has been paid to the function of circRNAs recently. A number of studies have verified that circRNAs are implicated in multiple human malignancies, including lung cancer [8–10]. Furthermore, recent researches have also revealed the critical effect of circRNAs exert on cellular sensitivity to cisplatin in different cancer types, such as osteosarcoma, gastric cancer and bladder cancer [11–13]. Therefore, identification of the circRNAs involved in regulating the sensitivity of lung cancer cells to cisplatin are of great value. Recently, existing literatures have uncovered the cancer-promoting role of circ-ABCB10 in clear cell renal cell carcinoma and breast cancer [14, 15]. However, the critical role of it in lung cancer and its association with cellular sensitivity to cisplatin in lung cancer are unclear, which therefore are worthy of exploring.

This study mainly focused on probing the regulatory mechanism of circ-ABCB10 and its influence on cell sensitivity to cisplatin in lung cancer. The results of this study elucidate that knockdown of circ-ABCB10 sensitized lung cancer cells to cisplatin via miR-556-3p/AK4 axis, which indicated that targeting circ-ABCB10 might be a new thought to improving the efficacy of cisplatin in lung cancer.

## Materials and methods

### Cell culture and treatment

Human bronchial epithelial cell (HBE) and human NSCLC cells (H-1299, H-125, NCI-H292, A549) were purchased from Chinese Academy of Sciences (Beijing, China). The RPMI-1640 medium (Invitrogen, Carlsbad, CA, USA) containing 10% fetal bovine serum (FBS; Invitrogen) and 1% penicillin/streptomycin (Sigma-Aldrich, Milan, Italy) was applied for culturing cells, and cells were cultured in an incubator with 5% CO_2_ at 37 °C. To study cellular sensitivity to drugs, 2 μg/ml of 5-fluorouracil (5-Fu), 1 μM of cisplatin, 10 μM of Sorafenib and 1 μM of Sunitinib were all utilized for treating A549 or NCI-H292 cells, all from Sigma-Aldrich. 0.1% DMSO (Sigma-Aldrich) was added to culture medium as a solvent-only negative control group.

### Cell transfection

A549 and NCI-H292 cells were transfected with specific shRNAs against circ-ABCB10 (sh-circ-ABCB10#1#2), AK4 (sh-AK4#1#2), negative control (sh-NC), pcDNA3.1/AK4 or the empty pcDNA3.1 vector (GenePharma, Shanghai, China), separately. The miR-556-3p mimics and NC mimics were gained from GenePharma. Each plasmid was transfected into cells by Lipofectamine 2000 (Invitrogen).

### Quantitative real time polymerase chain reaction (RT-qPCR)

TRIzol reagent (Takara, Otsu, Japan) was utilized for extracting total RNA from cultured A549 or NCI-H292 cells, and RNA concentration was measured with bicinchoninic acid (BCA) Kit (Invitrogen). Total RNA was reverse-transcribed into cDNA utilizing Reverse Transcription Kit (Applied Biosystems, Foster City, USA) for RT-qPCR. SYBR Green real-time PCR Kit (Takara, Tokyo, Japan) was then used on Bio-Rad CFX96 (Bio-Rad, Hercules, CA). Fold changes of gene expression were calculated by 2^−ΔΔCt^ method, with glyceraldehyde-3-phosphate dehydrogenase (GAPDH)/U6 as endogenous control.

### Actinomycin D (actD) and Rnase R treatment assays

2 mg/ml of actinomycin D (Sigma-Aldrich) was cultivated with A549 or NCI-H292 cells for 0, 4, 8, 12 h to block mRNA transcription, with DMSO as control. For treating cells with Rnase R, 2 μg of total RNA was cultivated for 30 min with or without 3 U/μg of Rnase R (Epicentre Technologies, Madison, WI, USA) at 37 °C, while cells without Rnase R treatment were seen as control group. RT-qPCR was employed for determining the expression levels of circABCB10 and ABCB10 mRNA.

### Nucleic acid gel electrophoresis assay

After amplifying ABCB10 mRNA and circ-ABCB10, the cDNA and genomic DNA (gDNA) were obtained as templates, their PCR products were assessed by the agarose gels with TE buffer (Thermo Scientific, Waltham, MA, USA). GAPDH was used as a negative control.

### CCK-8 assay

The A549 and NCI-H292 cells (1 × 10^4^) were inoculated into 96-well plates, and then incubated with 10 mg/ml of CCK-8 (Sigma-Aldrich) for another 4 h. The absorbance at 450 nm was measured with a plate reader (Bio-Tek Instruments, Hopkinton, MA, USA).

### Colony formation assay

1 × 10^3^ cells were cultured in 6-well plates and the culture medium was changed every 3 days. After 2 weeks, cells were fixed in ethanol (Sigma-Aldrich), and were then dyed using crystal violet (Sigma-Aldrich). Finally, the visible colonies containing ≥50 cells were then counted manually under a microscope (Olympus, Tokyo, Japan).

### TUNEL assay

Using TUNEL Apoptosis Kit (Invitrogen), the apoptosis of A549 and NCI-H292 cells were determined. The above cells were stained by utilizing DAPI (Koritai Biotechnology, Beijing, China). Then, the TUNEL positive cells were captured under a fluorescence microscopy (Olympus).

### Transwell assay

The migration abilities of 1 × 10^3^ A549 or NCI-H292 cells were examined using transwell chambers (Millipore, Billerica, MA, USA). Serum-free medium containing transfected cells was added to top compartment, while medium with 10% FBS was plated into bottom chamber. 48 h later, methanol (Sigma-Aldrich) and crystal violet (Solarbio, Beijing, China) were applied for fixing and dying cells, severally. 5 chosen fields at random under a microscope were used for counting migratory cells.

### Western blot

Total protein from cells were extracted by RIPA lysis buffer with protease inhibitors (Beyotime, Shanghai, China) and quantified through BCA Kit (Invitrogen). Then, total protein was isolated by SDS-PAGE and was moved to PVDF membranes (Millipore). After being blocked with skimmed milk, membranes were cultivated overnight with specific primary antibodies for AK4 (ab232888, Cambridge, USA) and GAPDH (ab8245). Later, secondary antibodies were added for cultivating for 1 h. The amount of protein was detected via chemiluminescence detection system.

### Subcellular fractionation

The fractions of cytoplasmic and nuclear were isolated via NE-PER™ Nuclear and Cytoplasmic Extraction Reagents (Invitrogen) and gathered by RNeasy Midi Kit (Qiagen, Hilden, Germany) to determine the cellular localization of circ-ABCB10. The RT-qPCR was used for exploring the expression levels of circ-ABCB10, U6 (nuclear control) and GAPDH (cytoplasmic control).

### FISH assay

Fluorescence-conjugated circ-ABCB10 probes were produced by Bersinbio Company (Guangzhou, China). NSCLC cells were immobilized in 4% paraformaldehyde (Sigma-Aldrich). After being cleaned using PBS and dehydrated in ethanol, the air-dried cells were denatured for further cultivation with FISH probes by hybridization reaction buffer. Later, cells were washed with × 2 saline-sodium citrate (SSC). Finally, DAPI was used for dying cells and results were visualized through a confocal laser microscopy (Olympus).

### RIP assay

RIP assay was completed using an Imprint RNA immunoprecipitation kit (Sigma-Aldrich). A549 and NCI-H292 cells were reaped and lysed in RIP lysis buffer. Cell lysate was then incubated with magnetic beads which were conjugated with anti-Ago2 antibody or with a negative control normal anti-IgG antibody. The relative enrichment of circ-ABCB10, miR-556-3p and AK4 were determined by RT-qPCR analysis.

### Luciferase reporter assay

The wild-type (WT) reporter vector psiCHECK-circ-ABCB10-WT (Promega, Madison, WI, USA) were constructed based on the bioinformatics-predicted binding sites of miR-556-3p, while binding sequences were mutated to form psiCHECK-circ-ABCB10-Mut vector. The two vectors were co-transfected with miR-556-3p mimics or NC mimics into A549 and NCI-H292 cells. The wild-type and mutant interacting sequences of miR-556-3p in AK4 3′-UTR fragments were obtained to generate psiCHECK-AK4-WT/Mut vectors, then co-transfected into cells with indicated transfection plasmids. The luciferase activity was examined at 48 h post-transfection via Dual-Luciferase Reporter Assay System (Promega, MA, USA).

### Statistical analysis

Statistical analyses were carried out by GraphPad Prism 7.0 software (Graph Pad Software, La Jolla, CA, USA). Data from more than two independent experiments, were showed as mean ± SD. Student’s t-test or one-way ANOVA was employed for the assessing group difference. *P* < 0.05 had statistical significance in requirements.

## Results

### Circular RNA circ-ABCB10 is highly expressed in NSCLC and circ-ABCB10 knockdown represses lung cancer progression and improves the sensitivity of lung cancer cells to cisplatin

**Fig. 1:**
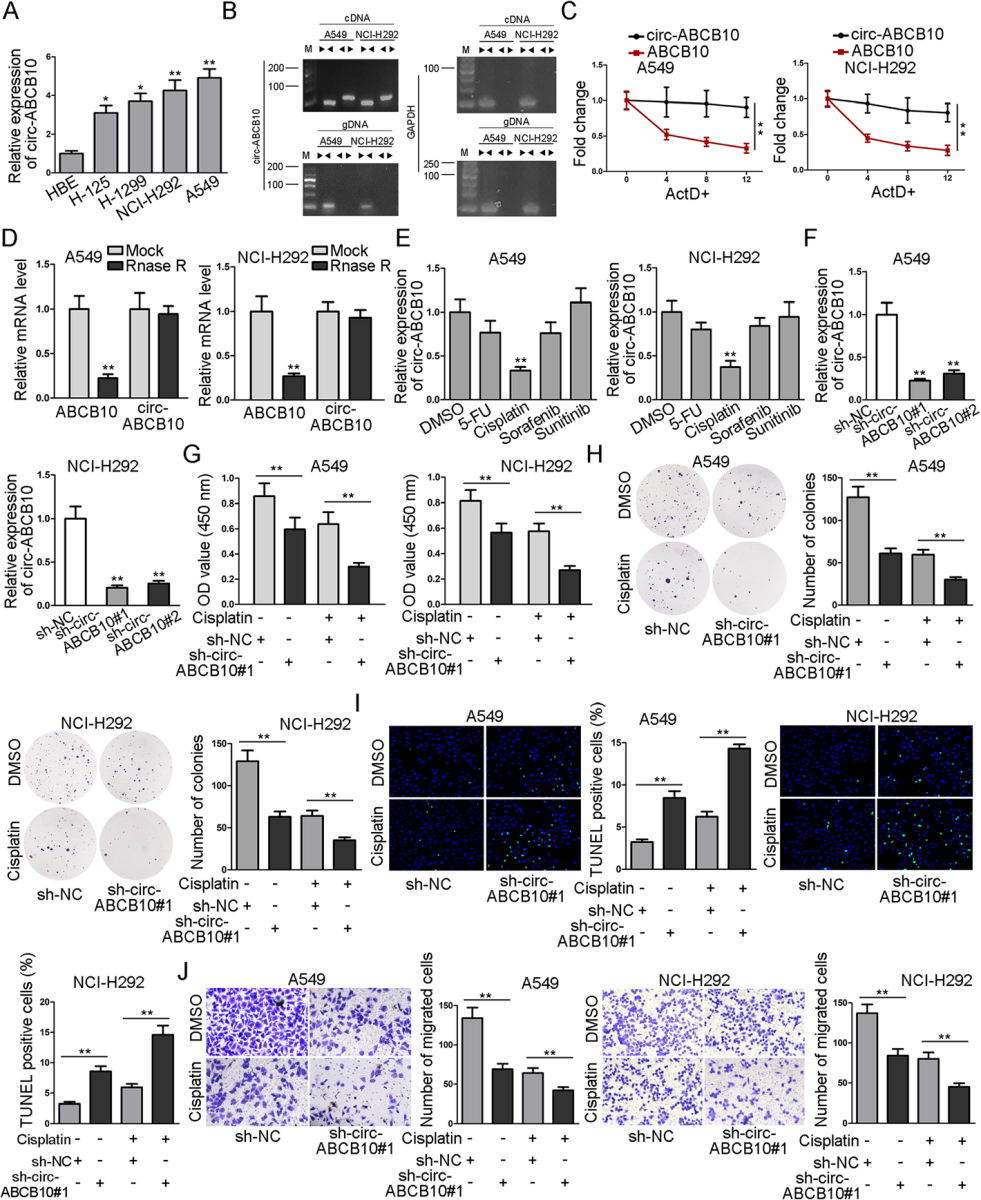
Circular RNA circ-ABCB10 is highly expressed and downregulation of it represses lung cancer progression and improves the sensitivity of lung cancer cells to cisplatin. **a** The expression of circ-ABCB10 in lung cells (H125, H1299, NCI-H292 and A549) and normal human bronchus epithelium cells (HBE) was detected via RT-qPCR. **b** Nucleic acid gel electrophoresis analysis revealed that divergent primers could produce the circular isoform of ABCB10 with cDNA but not with gDNA. GAPDH was used as a negative control. **c** The stability of circ-ABCB10 and ABCB10 mRNA was detected by RT-qPCR in A549 and NCI-H292 cells after treatment of ActD. **d** RT-qPCR assay was conducted to determine the abundance of circ-ABCB10 and linear ABCB10 mRNA in A549 and NCI-H292 cells treated with Rnase R (normalized to mock treatment). **e** The expression of circ-ABCB10 in A549 and NCI-H292 cells treated with different chemotherapy agents was determined via RT-qPCR analysis. **f** The efficiency of circ-ABCB10 knockdown in A549 and NCI-H292 cells was detected by RT-qPCR. **g**-**h** After treatment with or without cisplatin, the proliferation ability of transfected cells was measured by CCK-8 and colony formation assays. **i** TUNEL analysis of cell apoptosis ability in transfected cells with or without cisplatin treatment. **j** Transwell analysis of cell migration capability in transfected cells with or without cisplatin treatment. ^*^P < 0.05, ^**^P < 0.01

Previous studies have revealed the cancer-promoting role of circ-ABCB10 in clear cell renal cell carcinoma and breast cancer [14, 15]. Nevertheless, the critical role of it in lung cancer and its association with cisplatin resistance of lung cancer are kept to be explored. To begin with, we conducted RT-qPCR analysis and observed an abnormally high expression of circ-ABCB10 in lung cancer cell lines (H125, H1299, NCI-H292 and A549) compared with that in normal human bronchus epithelium cells (HBE) (Fig. 1a). Then, data from nucleic acid gel electrophoresis analysis revealed that divergent primers could produce the circular isoform of ABCB10 in complementary DNA (cDNA) but not genomic DNA (gDNA), whereas convergent primers were able to amplify the linear isoform of ABCB10 from both cDNA and gDNA in A549 and NCI-H292 cells (Fig. 1b). Under the treatment of ActD, the mRNA synthesis inhibitor, RT-qPCR results presented that ABCB10 mRNA degraded whereas circ-ABCB10 was stable within 12 h in A549 and NCI-H292 cells (Fig. 1c). Additionally, after treatment with Rnase R in A549 and NCI-H292 cells, ABCB10 expression was observably lowered whereas no obvious change of circ-ABCB10 expression could be observed (Fig. 1d). Subsequently, we aimed to explore whether dysregulation of circ-ABCB10 was implicated in the sensitivity of lung cancer cells to cisplatin. As illustrated in Fig. 1e, in comparison with that in control cells, the expression of circ-ABCB10 was significantly declined in cisplatin-treated cells rather than other drugs including 5-FU, Sorafenib, and Sunitinib, indicating that circ-ABCB10 was related to cellular response to cisplatin in lung cancer. To uncover the effect of circ-ABCB10 on cellular sensitivity to cisplatin and lung cancer progression in vitro, we knocked down circ-ABCB10 in NCI-H292 and A549 cells. RT-qPCR depicted a better satisfactory efficiency of circ-ABCB10 knockdown in A549 and NCI-H292 cells by transfection with sh-circ-ABCB10#1 (Fig. 1f). Next, CCK-8 and colony formation assays depicted that compared with that sh-circ-ABCB10#1 strikingly weakened the proliferation ability of lung cancer cells compared with sh-NC group. Cisplatin treatment abrogated lung cancer cell proliferation, and such effect was further enhanced by sh-circ-ABCB10#1 in A549 and NCI-H292 cells were treated with cisplatin (Fig. 1g-h). Moreover, TUNEL assay testified that cell apoptosis capability in sh-circ-ABCB10#1 group was significantly increased when compared with sh-NC group. The treatment of cisplatin increased apoptosis ratio, such increase was further enhanced in sh-circ-ABCB10#1 + cisplatin group (Fig. 1i). Furthermore, transwell assay demonstrated that the migration ability was weakened in lung cancer cells transfected with sh-circ-ABCB10#1 compared with sh-NC control. Cisplatin treatment also decreased migrated cells, and sh-circ-ABCB10#1 could strengthen the effect of cisplatin (Fig. 1j). In sum, circ-ABCB10 is expressed at high levels and featured with circular structure in lung cancer; circ-ABCB10 deletion represses lung cancer progression whereas sensitizes lung cancer cells to cisplatin.

### Circ-ABCB10 sponges miR-556-3p in lung cancer

**Fig. 2:**
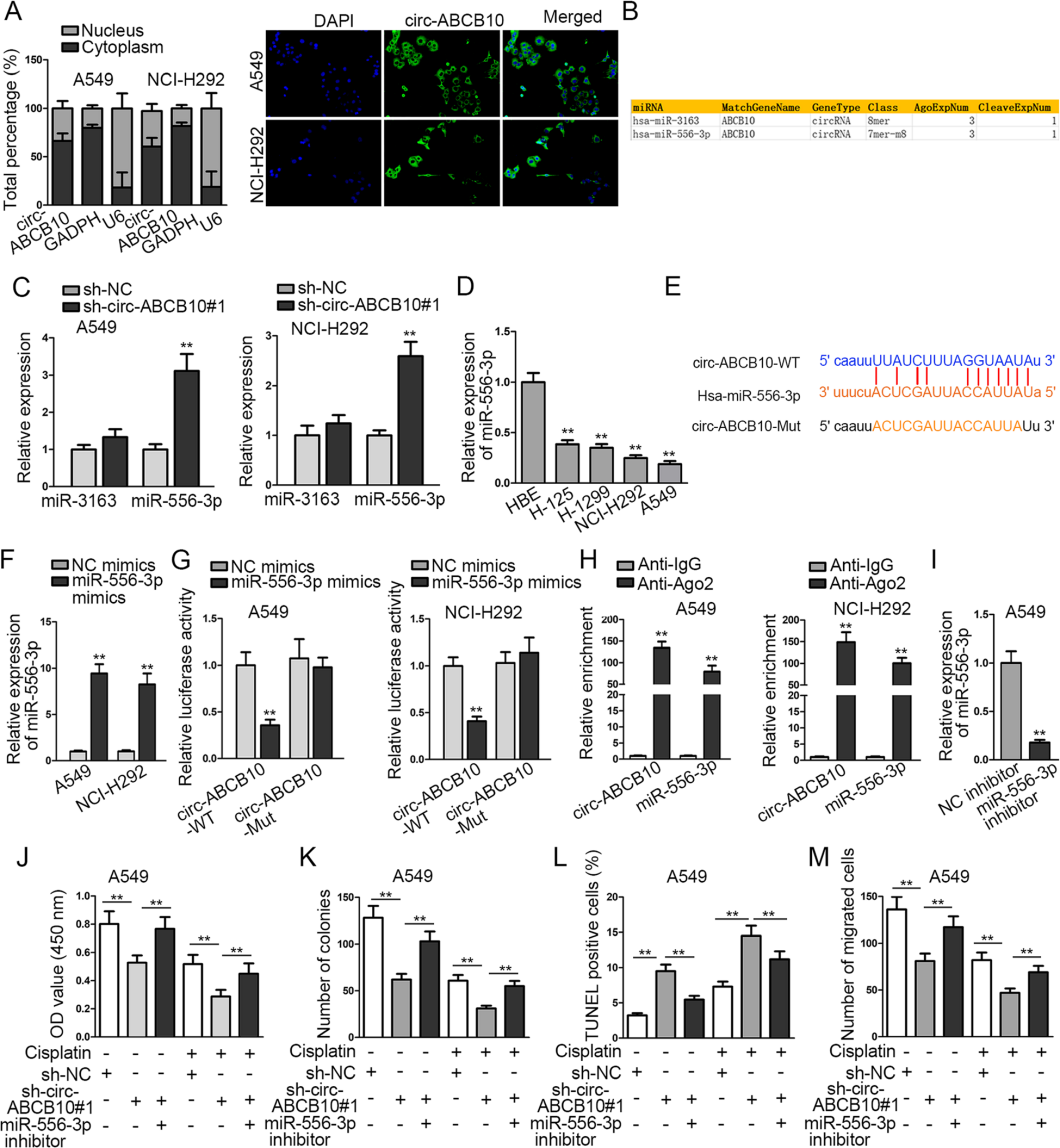
Circ-ABCB10 sponges miR-556-3p in lung cancer. **a** The subcellular localization of circ-ABCB10 was detected by subcellular fractionation and FISH assays. **b** MiRNAs that could bind with circ-ABCB10 were predicted through starBase. **c** RT-qPCR analysis of miR-556-3p expression in transfected cells. **d** RT-qPCR analysis of miR-556-3p expression in lung cancer cells and HBE cells. **e** A binding site between circ-ABCB10 and miR-556-3p was predicted via starBase. **f** The efficiency of miR-556-3p overexpression was analyzed via RT-qPCR. **g**-**h** The interaction between circ-ABCB10 and miR-556-3p was testified by luciferase reporter and RIP assays. **i** The efficacy of miR-556-3p inhibition was analyzed via RT-qPCR. **j**-**m** Cell proliferation, apoptosis and migration in different groups were separately evaluated by CCK-8, colony formation, TENEL and transwell assays. ^**^P < 0.01

To investigate the underlying mechanism of circ-ABCB10 in lung cancer, we carried out subcellular fractionation and FISH assays to investigate the localization of circ-ABCB10. Results showed that circ-ABCB10 was mainly distributed in cytoplasm of A549 and NCI-H292 cells (Fig. 2a). Thus, we suspected that circ-ABCB10 played vital role in lung cancer via sponging specific miRNA. Subsequently, under the particular screening condition (CLIP Data: strict stringency > = 3; Degradome Data: low stringency > = 1) in starBase (http://starbase.sysu.edu.cn/), miRNAs (miR-3163 and miR-556-3p) that was predicted to bind with circ-ABCB10 were listed in Fig. 2b. Then, we further find out the miRNA related to circ-ABCB10 in lung cancer. Data from RT-qPCR analysis showed that miR-556-3p, rather than miR-3163, was upregulated by sh-circ-ABCB10#1 in A549 and H292 cells compared with sh-NC control (Fig. 2c). Thus, we focused on investigating miR-556-3p. We delineated via RT-qPCR that miR-556-3p expression was significantly decreased in lung cancer cells versus the normal HBE cells (Fig. 2d). In addition, a binding site between circ-ABCB10 and miR-556-3p was predicted via starBase (Fig. 2e). After transfection with miR-556-3p mimics, miR-556-3p expression was conspicuously elevated in A549 and NCI-H292 cells (Fig. 2f). Luciferase reporter assay displayed that forced expression of miR-556-3p led to an obvious decrease in the luciferase activity of psiCHECK-circ-ABCB10-WT whereas caused no distinct alteration on the luciferase activity of psiCHECK-circ-ABCB10-Mut (Fig. 2g). It was revealed through RIP analysis that circ-ABCB10 and miR-556-3p were notably enriched in anti-Ago2 group (Fig. 2h). To further verify the effect that the circ-ABCB10 regulated lung cancer progression and sensitivity to cisplatin via interacting with miR-556-3p, we applied rescue assays in transfected cells treated with or without cisplatin. RT-qPCR confirmed the significant inhibition of miR-556-3p expression by miR-556-3p inhibitor (Fig. 2i). As displayed in Fig. 2j-m, miR-556-3p inhibitor reversed the effect of circ-ABCB10 knockdown on inhibiting cell proliferation, inducing apoptosis, and retarding migration. Also, miR-556-3p counteracted the sensitizing effect of sh-circ-ABCB10#1 on cellular response to cisplatin (Fig. 2 j-m). Taken together, circ-ABCB10 directly interacts with miR-556-3p to regulate proliferation, migration, apoptosis, and sensitivity to cisplatin in lung cancer cells.

### Circ-ABCB10 regulates AK4 expression by sponging miR-556-3p in lung cancer

**Fig. 3:**
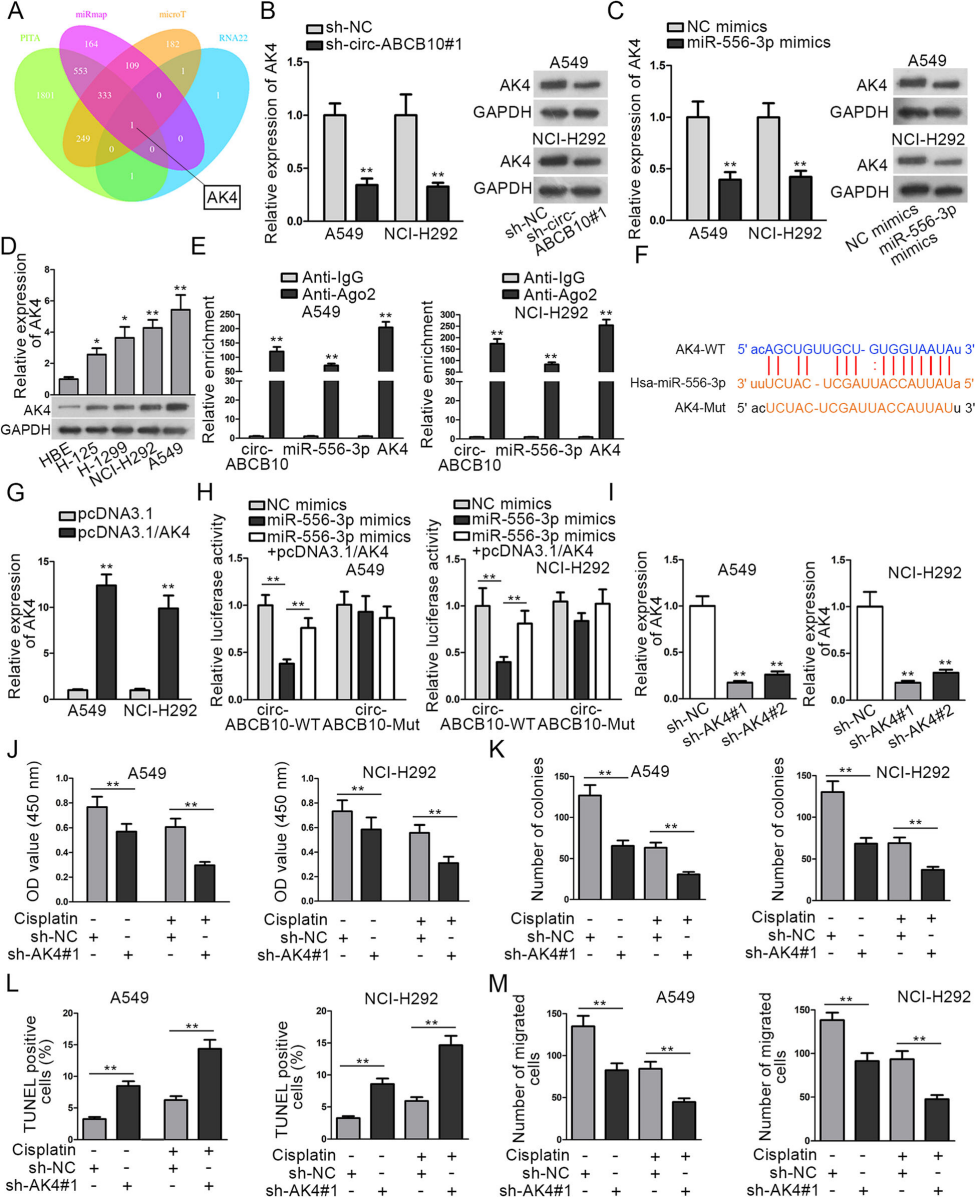
Circ-ABCB10 regulates AK4 expression by sponging miR-556-3p in lung cancer. **a** Venn pattern showed the overlap of the prediction results of four databases. **b**-**c** AK4 expression in transfected cells were examined through RT-qPCR together with western blot. **d** The expression of AK4 in lung cells and HBE cells was observed via RT-qPCR and western blot. **e** Measurement of RNA enrichments through RIP and RT-qPCR. **f** A binding site between miR-556-3p and AK4 predicted by starBase was shown. **g** The efficiency of AK4 overexpression was analyzed via RT-qPCR. **h** The interaction between RNAs was confirmed by luciferase reporter assay. **i** The efficiency of AK4 knockdown was analyzed via RT-qPCR. **j**-**m** Cell proliferation, apoptosis and migration in different groups were separately evaluated by CCK-8, colony formation, TENEL and transwell assays. ^*^P < 0.05, ^**^P < 0.01

In subsequence, we attempted to screen out the target mRNA of miR-556-3p. Through searching PITA, miRmap, microT and RNA22 databases, AK4 was predicted to be the target gene of miR-556-3p (Fig. 3a). Then RT-qPCR and western blot revealed that silencing circ-ABCB10 or overexpressing miR-556-3p could decrease the expression of AK4 in A549 and NCI-H292 cells (Fig. 3b-c). Additionally, a prominently elevated expression of AK4 in lung cells was observed via RT-qPCR and western blot (Fig. 3d). Later on, RIP assay depicted that circ-ABCB10, miR-556-3p and AK4 were all aggregated in the immunoprecipitates of anti-Ago2, which revealed the coexistence of them in RNA-induced silencing complex (RISC) (Fig. 3e). Thereafter, a binding site between miR-556-3p and AK4 predicted by starBase was presented in Fig. 3f. After transfection with pcDNA3.1/AK4, the expression of AK4 in A549 and NCI-H292 cells was remarkably increased (Fig. 3g). Afterwards, data from luciferase reporter assay uncovered that the reduced luciferase activity of psiCHECK-circ-ABCB10-WT resulted from overexpression of miR-556-3p could be recovered by overexpressing AK4. However, the luciferase activity of psiCHECK-circ-ABCB10-Mut exhibited no clear changes in different groups (Fig. 3h). Then, we studied the potential influence of AK4 on lung cancer cell progression and sensitivity to cisplatin. First, we adopted RT-qPCR analysis to detect the efficiency of AK4 knockdown. As shown in Fig. 3i, AK4 expression was decreased most significantly in sh-AK4#1-transfected cells. Subsequently, cell proliferation and apoptosis assays illuminated that compared with sh-NC group, sh-AK#1 reduced proliferation and induced apoptosis in A549 and NCI-H292 cells. Also, the anti-proliferation and pro-apoptosis effects of cisplatin in two lung cancer cell lines were further strengthened by sh-AK#1 (Fig. 3j-l). Additionally, transwell assay demonstrated that the migration ability of lung cancer cells in sh-AK4#1 group was markedly lower than that in sh-NC group, and the migration retarded by cisplatin was further decreased by sh-AK4#1 in lung cancer cells (Fig. 3m). Briefly, AK4 is a downstream target of miR-556-3p and its depletion inhibits lung cancer progression and sensitizes lung cancer cells to cisplatin.

### Circ-ABCB10 contributes to cisplatin resistance of lung cancer by targeting miR-556-3p/AK4 axis

**Fig. 4:**
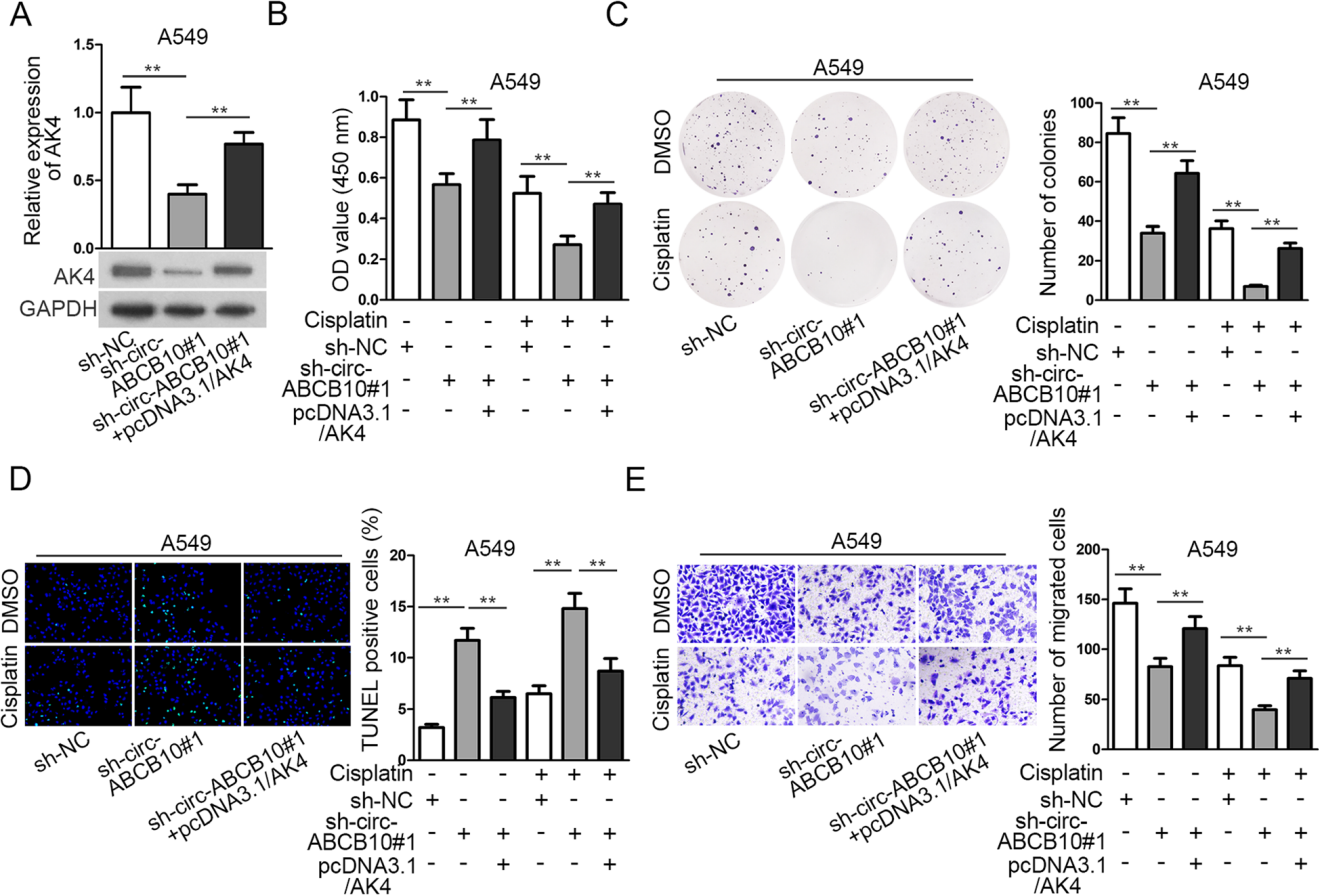
Circ-ABCB10 contributes to cisplatin resistance of lung cancer by targeting miR-556-3p/AK4 axis. **a** The mRNA and protein expressions of AK4 in A549 cells transfected with different plasmids were detected by RT-qPCR and western blot. **b**-**e** CCK-8, colony formation, TENEL and transwell assays were performed respectively to analyze cell proliferation, apoptosis and migration in different groups. ^**^P < 0.01

Based on the above findings, we intended to validate the role of circ-ABCB10/miR-556-3p/AK axis in regulating lung cancer progression and cell sensitivity to cisplatin. According to the data from RT-qPCR and western blot, the expression of AK4 was cut down by silencing circ-ABCB10 and then AK4 expression was restored by overexpressing AK4 (Fig. 4a). Then CCK-8, colony formation and TUNEL assays validated that upregulation of AK4 reversed the effect of circ-ABCB10 depletion on cell decreasing proliferation and increasing apoptosis (Fig. 4b-d). Knockdown of circ-ABCB10#1 strengthened the anti-proliferation and pro-apoptosis effects of cisplatin on A549 cells, and such strengthening effect of circ-ABCB10#1 was counteracted by AK4 overexpression (Fig. 4b-d). Similarly, upregulating AK4 expression could offset the suppressive effect of circ-ABCB10 knockdown on the migration of A549 cells treated without cisplatin (Fig. 4e). The sh-circ-ABCB10#1 enhanced the suppressive effect of cisplatin on migration of A549 cells, and such enhancement was countervailed by AK4 overexpression (Fig. 4e). To be concluded, circ-ABCB10 weakened the cellular sensitivity to cisplatin via miR-556-3p/AK4 axis in lung cancer.

## Discussion

Due to the highest incidence and death rate globally, lung cancer is diagnosed as the most prevalent malignancy [1]. Although cisplatin has been recently used as a kind of anti-cancer chemotherapy agent, multiple cancers, including lung cancer, may develop the acquired resistance to cisplatin [6]. Therefore, a further understanding of the mechanism behind the cellular sensitivity of lung cancer cells might help identify potential target to enhance cisplatin efficacy in lung cancer.

A growing number of researches have revealed the critical function of circRNAs on cisplatin resistance of diverse cancers [11–13]. Therefore, identification and exploration of the circRNAs associated with cisplatin efficacy are of great value. Previous findings showed that circ-ABCB10 was upregulated and served as an oncogene in several cancers such as clear cell renal cell carcinoma and breast cancer [14, 15]. Also, a study by Tian X. et al. validated that circ-ABCB10 promoted the proliferation and migration of non-small cell lung cancer (NSCLC) [16]. In consistence, our data also validated that circ-ABCB10 was upregulated in non-small cell lung cancer cell lines, and that knockdown of circ-ABCB10 decreased proliferation, increased apoptosis, and hindered migration in NSCLC cells. Notably, our data firstly showed that cisplatin treatment led to a decrease of circ-ABCB10 in lung cancer cells, indicating the involvement of circ-ABCB10 in the cellular response to cisplatin in lung cancer. This was the first time for circ-ABCB10 to be related to drug sensitivity in cancer cells. Further, we demonstrated that knockdown of circ-ABCB10 can further enhance the anti-proliferation and pro-apoptosis effect of cisplatin on lung cancer cells, suggesting that targeting circ-ABCB10 sensitized lung cancer cells to cisplatin in vitro.

Mechanistically, increasing evidence has indicated that circRNAs sponge specific miRNAs to regulate tumor initiation and progression [17, 18]. Several studies also demonstrated that circ-ABCB10 can sponge miRNAs to regulate certain oncogenes in cancers including NSCLC [15, 16]. Concordantly, we showed that circ-ABCB10 was mainly distributed in cytoplasm of lung cancer cells, verifying that circ-ABCB10 played vital roles in lung cancer by post-transcriptionally regulating certain miRNA. We firstly predicted through bioinformatics tool and validated that miR-556-3p interacted with circ-ABCB10. Formerly, miR-556-3p has been reported to be lowly expressed in endometrial tissues [19]. However, the association of miR-556-3p with circ-ABCB10 was first confirmed by our data. Also, we validated via rescue assays that miR-556-3p participated in the regulation of circ-ABCB10 on lung cancer progression and sensitivity to cisplatin resistance of lung cancer cells, which also firstly linked miR-556-3p to lung cancer and drug sensitivity of lung cancer cells.

Adenylate kinase 4 (AK4), has been validated to exert oncogenic function on lung cancer cell growth and metastasis [20]. Besides, AK4 has been uncovered to be involved in the radio-resistance of esophageal cancer cells [21]. More importantly, previous investigations have confirmed the involvement of AK4 in chemoresistance of cancers [22, 23]. Formerly, a study suggested the correlation of high AK4 level with poor survival in lung cancer [24], but the detailed role of AK4 in lung cancer progression and cell response to cisplatin was first revealed in our study. Herein, we validated that AK4 was highly expressed in lung cancer cells. We also confirmed that AK4 was negatively regulated by miR-556-3p but positively regulated by circ-ABCB10. Besides, we validated that circ-ABCB10 competed with AK4 to bind to miR-556-3p, which suggested that circ-ABCB10 sponged miR-556-3p to upregulate AK4 by serving as a ceRNA. Moreover, downregulation of AK4 restrained lung cancer progression whereas sensitizes lung cancer cells to cisplatin. Finally, rescue assays manifested that upregulating AK4 expression could offset the suppressive effect of circ-ABCB10 knockdown on the progression of lung cancer cells without cisplatin, and that AK4 overexpression counteracted the sensitizing effect of circ-ABCB10 knockdown on cells to cisplatin treatment, indicating that AK4 was a target for circ-ABCB10 to regulate lung cancer progression and cell sensitivity to cisplatin.

## Conclusion

In summary, all these data from this study indicate that targeting circ-ABCB10/miR-556-3p/AK4 pathway enhance sensitivity of lung cancer cells to cisplatin. These findings suggested that targeting circ-ABCB10 might be a potential new thought to improve the sensitivity to cisplatin in lung cancer. However, studies based on clinical samples and in vivo models are required to validate the clinical significance of circ-ABCB10, which we will carry out in the future.

## Data Availability

The datasets involved in the present study are available from the corresponding author upon reasonable request.

## Abbreviations

ActD: Actinomycin D
AK4: Adenylate kinase 4
CeRNA: Competing endogenous RNA
CircRNAs: Circular RNAs
LncRNAs: Long noncoding RNAs
MiRNAs: MicroRNAs
NcRNAs: Noncoding RNA
RISC: RNA-induced silencing complex
